# Development-related alternative splicing of PAC1 receptor: a key player in schizophrenia?

**DOI:** 10.3389/fnmol.2013.00021

**Published:** 2013-08-12

**Authors:** Maurício Rocha-Martins, Brian Njaine

**Affiliations:** Neurobiology Department, Institute of Biophysics Carlos Chagas Filho, Federal University of Rio de JaneiroRio de Janeiro, Brazil

Alternative splicing is the process by which various combinations of exons are included in a mature mRNA, thus allowing a single gene to encode multiple protein isoforms with potentially different or even antagonistic properties. Proper mRNA splicing is absolutely crucial for nervous system development and its misregulation has been demonstrated in schizophrenia (Morikawa and Manabe, 2010). Schizophrenia is a severe and debilitating neuropsychiatric syndrome which has a complex etiology and is believed to arise from the interplay between genetic and environmental factors. Altered expression of genes involved in early migration of neurons and glia, cell proliferation, axonal outgrowth and synaptogenesis in schizophrenic patients support a neurodevelopmental model.

A transcriptome sequencing study of the cortex of schizophrenic patients recently revealed significant differences in PAC1 receptor alternative splicing (Wu et al., 2012), but the functions of the aberrant isoforms are still unclear. PAC1 is a G-protein-coupled receptor activated by pituitary adenylate cyclase activating polypeptide (PACAP, *Adcyap1*), which is a potential schizophrenia susceptibility gene and whose loss of function in mice (PACAP^−/−^) showed several schizophrenia-related behaviors (Hashimoto et al., 2007).

PACAP acts as neurotransmitter and/or neurotrophic peptide via PAC1 (or VPAC receptors) with a wide range of functions throughout ontogenesis (e.g., proliferation and survival control). In rat, eight splice variants of the PAC1 receptor have been identified that mainly trigger adenylate cyclase (AC) and/or phospholipase C (PLC) activation.

The description of PAC1 receptor isoforms shed light on the unresolved pleiotropic biological functions of PACAP. In a recent paper in The Journal of Neuroscience, Yan et al. (2013) addressed this question in the context of cortical neurogenesis and provided compelling evidence that developmental switch of PAC1 receptor mRNA isoforms accounts for both pro- and anti-mitogenic actions of PACAP.

Previous studies indicated that PACAP signaling inhibits the proliferation of cortical precursor cells from E13.5 onward. In contrast, they initially found that PACAP^−/−^ mice exhibit reduced S-phase labeling at E9.5 ventricular zone (VZ). Then, Yan et al. (2013) developed a novel culture model of young neural precursors that was instrumental to define the contribution of PAC1 receptor variants on PACAP proliferative effects on early neurogenesis.

Using this system, they assessed the expression and function of PAC1 short isoform and insert-containing hop isoform (PAC1 hop). These isoforms are coupled to different transduction pathways (AC and AC/PLC, respectively) and were previously shown to elicit antagonist effects in cortical precursors proliferation. Specific pairs of primers designed for each PAC1 isoform showed a 24-fold greater content of hop isoform mRNA at E10.5, whereupon PACAP exposure promoted G1 cyclins and G1/S-phase progression. In contrast, by E14.5, in which PACAP exposure promoted cell cycle exit, short isoform mRNA content was 15-fold greater than hop.

Two intriguing questions, then, may be formulated: (1) which RNA binding proteins (splicing activators or repressors) regulate the developmental switch in the expression of PAC1 isoforms?, and (2) Are these RNA-binding proteins misregulated in neurodevelopmental disorders? Interestingly, A2BP1, a sequence-specific RNA-binding protein, could be a starting point to answer these questions, as it promotes the generation of PAC1-hop mRNA isoform (Amir-Zilberstein et al., 2012). Moreover, A2BP1 is differentially expressed throughout cortical development and *de novo* copy number mutations of this gene contribute to the genetic component of schizophrenia (Melhem et al., 2011).

In addition, using a shRNA specific to PAC1hop, the authors further demonstrated that PACAP pro-mitogenic effect requires PAC1hop expression. The general notion is that upon PACAP binding to PAC1hop, the PLC transduction pathway is activated and two second messengers are then generated. Diacylglycerol (DAG) leads to PKC activation, whereas inositol triphosphate (IP3) triggers calcium (Ca^2+^) release from endoplasmic reticulum stores. Both stimuli are well-known stimulators of cell proliferation in multiple systems. Consistently, a PLC-specific inhibitor blocked PACAP pro-mitogenic effect.

It is noteworthy that PACAP was able to trigger Ca^2+^ influx and PKC activation only at E10.5, but not at E14.5, even though PAC1hop expression changed little between these stages. In conclusion, the authors showed that the proper balance between PAC1-short and -hop isoforms determines the activation of PLC and consequently the pro- or anti-mitogenic actions of PACAP signaling.

Overall, the study by Yan et al. (2013) in combination with other recent reports has provided novel insights into how misregulation of PAC1 alternative splicing could contribute to the risk of developing schizophrenia, not only by affecting embryonic neurogenesis, but also by impairing proliferation and differentiation of adult stem cells. Accordingly, Reif et al. (2006) demonstrated a reduction in the amount of hippocampal neural stem cells in post-mortem schizophrenic brains (Reif et al., 2006), raising the idea that this could explain the reduction of hippocampal volume and the correlation with several cognitive deficits. PAC1 is also expressed in the classical neurogenic regions of the adult mouse brain, namely the VZ of the lateral ventricle and the hippocampal dentate gyrus, and its activation promotes neural stem cell proliferation (Mercer et al., 2004).

Although significant progress has been made toward the understanding of how PACAP and PAC1 contributes to the pathogenesis of schizophrenia, little is known about the expression and function of PAC1 isoforms in the cortex and in the neurogenic regions of patients. Moreover, the concept that developmental transition in the expression of PAC1 splice isoforms leads to PACAP antagonistic effects on neurogenesis suggests a possible compensation mechanism that could mask more severe symptoms caused by deregulated neurogenesis in PACAP knockout mice. Therefore, development of transgenic animal models to mimic alterations in PAC1 alternative splicing found in schizophrenia would help to unveil a possible association between PACAP-PAC1 signaling and schizophrenia.
